# Comparison of different comorbidity measures for use with administrative data in predicting short- and long-term mortality

**DOI:** 10.1186/1472-6963-10-140

**Published:** 2010-05-27

**Authors:** Yu-Tseng Chu, Yee-Yung Ng, Shiao-Chi Wu

**Affiliations:** 1 Institute of Health and Welfare Policy, School of Medicine, National Yang-Ming University, Taipei, Taiwan; 2 Department of Medicine, Taipei Veterans General Hospital, Taipei, Taiwan; 3 Department of Medicine, School of Medicine, National Yang-Ming University, Taipei, Taiwan

## Abstract

**Background:**

It is important to find a comorbidity measure with better performance for use with administrative data. The new method proposed by Elixhauser et al. has never been validated and compared to the widely used Charlson method in the Asia region. The objective of this study was to compare the performance of three comorbidity measures using information from different data periods in predicting short- and long-term mortality among patients with acute myocardial infarction (AMI) and chronic obstructive pulmonary disease (COPD).

**Methods:**

We conducted a retrospective cohort study using National Health Insurance claims data (2001-2002) in Taiwan. We constructed the Elixhauser, the Charlson/Deyo, and the Charlson/Romano methods based on the International Classification of Disease, 9th Revision, Clinical Modification codes in the claims data. Two data periods, including the index hospitalization as well as the index and prior 1-year hospitalizations, were used in the analysis. The performances were compared using the c-statistics derived from multiple logistic regression models that included age, gender, race, and whether the patient received surgery or not. The outcomes of interest were in-hospital and 1-year mortality.

**Results:**

The performance was in the same rank order among both populations regardless of the outcome and data period: Elixhauser > Charlson/Romano > Charlson/Deyo. In predicting in-hospital mortality, the Elixhauser models using information from the index hospitalization performed best, even better than the Charlson/Deyo or Charlson/Romano models using information from the index and prior hospitalizations. Nevertheless, in predicting 1-year mortality, the Elixhauser models using information from the index and 1-year prior hospitalizations performed better than using information from the index hospitalization only.

**Conclusions:**

This is so far the first study to validate the Elixhauser method and compare it to other methods in the Asia region, and is the first to report its differences in data periods between short- and long-term outcomes. The comorbidity measurement developed by Elixhauser et al. has relatively good predictive validity, and researchers should consider its use in claims-based studies.

## Background

Administrative databases are increasingly used in health services research, epidemiologic research, and outcome studies. For control of baseline differences in these observational data, one major issue is to find a comorbidity measure with better performance [1-9]. Evidence from different population and datasets are needed for the generalizability of comparative performance [6,10]. However, there are very limited studies in Asian countries.

Among various methods, the Charlson comorbidity index (CCI) has been used most widely. It was developed by considering the impact of comorbidities in predicting 1-year mortality of medical inpatients using comorbidity data recorded in the medical charts [11]. Two adaptations for use with International Classification of Disease, 9^th ^Revision, Clinical Modification (ICD-9-CM) codes in administrative databases were made by Deyo et al.[12] (Charlson/Deyo) and Romano et al.[13] (Charlson/Romano), both of which defined 17 comorbidity categories. These two methods distinguish some comorbidities from complications by identifying whether the diagnosis codes appear in the admissions prior to the index hospitalization [12,13]. The Charlson/Deyo method is used most widely in the world including Taiwan [6], however, it has not been compared to other methods in Asian countries. In addition, the Charlson/Romano method has been evaluated to be better or no different from the Charlson/Deyo method in the United States [13-17].

Little literature showed that the new method proposed by Elixhauser et al.[18] had better performance than the Charlson/Deyo method in predicting in-hospital mortality[6,7]. The Elixhauser method, which includes 30 categories of comorbid conditions, was developed using administrative data and was reported with the ability to predict length of stay, hospital charges, and in-hospital death [18]. The comorbid conditions were considered only when they did not relate to the diagnosis-related group (DRG) of each admission, therefore no prior admission was needed to distinguish between comorbidities and complications [18]. However, evidence of the Elixhauser method's performance from different populations and datasets is still relatively scarce. It also has not been compared to other methods in Asian countries.

The Charlson based methods was developed earlier than the Elixhauser method. It had already been widely used before its adaptation to the administrative data. The Elixhauser method uses both ICD-9-CM codes and Diagnosis-Related Groups (DRGs), and thus is more complicated to use. The Elixhauser method has more variables than the Charlson based methods, and therefore needs larger sample size. However, the increasingly used administrative databases make sample size large enough for analysis.

Factors influencing the performance of claims-based comorbidity measures include the dataset, studied population, outcome, and data periods [7,19]. Comparative performance can be examined for one factor, while other factors are held constant [4,17]. Although several published studies have investigated the relative performance of various claims-based comorbidity measures and factors influencing performance, evidence from the Asian region or different administrative datasets is still lacking. Furthermore, since different outcomes were concerned by Charlson et al.[12] and Elixhauser et al.[18] (1-year mortality vs. in-hospital mortality), and different data periods in claims data were used (index and prior hospitalizations vs. index hospitalization only), little is known about the effect of the combination of these factors. Therefore, the purpose of this study is to compare the performance of three claims-based comorbidity measures, the Elixhauser, the Charlson/Deyo, and the Charlson/Romano methods, using National Health Insurance claims data in Taiwan. We also assessed the relative performance of the three methods when using different data periods to predict in-hospital mortality and 1-year mortality.

## Methods

### Data Sources

Data are collected from the National Health Insurance inpatient claims data, family registration file, and death certification data. An encrypted unique identification number is used to link information of the same patient from different datasets.

Taiwan had a universal single-payer National Health Insurance Program since 1995. As of 2002, 97.1% of Taiwan's population was enrolled in this program [20]. All contracted providers must regularly submit claim information to get reimbursement. Large computerized databases derived and managed by the Bureau of National Health Insurance are provided to scientists in Taiwan for research purposes (http://www.nhi.gov.tw/; http://w3.nhri.org.tw/nhird//index.php). The inpatient claims data include the dates of admission and discharge, sex, birth date, and diagnosis and procedure codes using the International Classification of Disease, 9th revision, Clinical Modification (ICD-9-CM). Each inpatient record includes the principle and up to 4 secondary diagnoses as well as 3 procedures.

The family registration file is used to identify the race of the patients (whether the patients are aboriginals or not). This database is maintained by the government and provides relatively accurate demographic information on residents (http://www.ris.gov.tw/). The database of death certificates, managed by the Department of Health in Taiwan, is a national registry of all deaths in Taiwan (http://www.doh.gov.tw/).

### Study Populations

The two study populations are inpatients with a principal diagnosis in the following two disease categories: acute myocardial infarction (AMI) (ICD-9-CM: 410.x) and chronic obstructive pulmonary disease (COPD) (ICD-9-CM: 490.x, 491.x, 492.x, 494.x, 496.x). We used the Clinical Classifications Software (CCS), which is published and freely downloadable from the website of the Agency for Healthcare Research and Quality (AHRQ) (http://www.hcup-us.ahrq.gov[21]), to define each disease category. These two disease categories were selected because they provide common acute and chronic conditions. We identified the first hospitalizations of cases between 1st January 2002 and 31st December 2002 as the index hospitalizations, and excluded patients younger than 18 years old and those who were not discharged before 31st December 2002. There were 8,961 AMI and 32,755 COPD patients who met the criteria.

### Data Periods

There were two data periods used in our analysis. One is the index hospitalization. The other one is the index and prior 1-year hospitalizations. The index and prior 1-year hospitalizations had 1-year lookback period. The index hospitalization didn't have lookback period. The index hospitalization was identified as the first hospitalization of our study population during year 2002. The index and prior 1-year hospitalizations included the index hospitalization and all hospitalizations 1-year before the index date.

### Outcomes of Interest

In-hospital all-cause mortality and 1-year all-cause mortality were selected because they represented short- and long-term outcomes. The administrative claims data was linked to death certification data to identify when the patient died.

### Comorbidity Measures

We compared three published claims-based comorbidity measures [12,13,18]. Two of them are different ICD-9-CM adaptations of the Charlson method [11], the Charlson/Deyo method [12] and the Charlson/Romano method [13]. Since there is no scoring system of the original Elixhauser method [18,22], comorbidity variables are created as individual categories (the presence or absence of the comorbidity) of three methods, and findings can be compared to the previous study which specified the same approach[6].

The third comorbidity method is developed by Elixhauser et al. using administrative data from California [18]. We identified comorbid conditions in the Elixhauser method using both ICD-9-CM codes and Diagnosis-Related Groups (DRGs). The definition codes of the Elixhauser method are updated and publicly available from the Healthcare Cost and Utilization Project (http://www.ahcpr.gov/data/hcup/comorbid.htm[23]), and we mapped its DRG codes to Taiwan's version of DRG codes [24].

### Analysis

Two data periods, including the index hospitalization as well as the index and prior 1-year hospitalizations, were used to predict in-hospital and 1-year mortality in the AMI and COPD inpatients. Variables in the baseline model included age, sex, race (aborigines vs. non-aborigines), and whether the patient received surgery or not. The aboriginality is an important risk factor for health outcomes [25,26]. We used surgical DRGs to identify whether a patient received surgery, as specified by Elixhauser et al. [7,18]. Each of the comorbidity measures was added to the baseline model. Additional file 1 provides a visual comparison of components of the models.

The statistical performance of each model was assessed using logistic regression [9]. Three comparisons were made (1) to the baseline model, (2) across comorbidity measures for the same outcome, population, and data period, and (3) within the same outcome, population, and comorbidity measure, but across data periods.

To assess whether adding the comorbidity measure improved the fit of the regression models, we reported G^2 ^statistics, which resulted from comparing the maximized likelihood function of a model including the comorbidity variables with the nested baseline model. The model comparison statistic has a Chi-squared distribution with the degree of freedom (df) equal to the difference in number of parameters [27].

To evaluate model discrimination, we reported c statistics, which represents the area under the receiver-operating characteristic (ROC) curve. The c-statistic values range from 0.5 (no greater predictive power than chance) to 1.0 (perfect prediction). Finally, bootstrapping with 1000 replications was conducted to obtain the 95% confidence interval of the c-statistics. This percentile-based confidence interval evaluates the variability of the c-statistic [5,28]. All analyses were performed using SAS version 9.1.3 (SAS Institute Inc, Cary, NC) [29].

## Results

We studied 8,961 AMI and 32,755 COPD patients. The proportion of the males and the aborigines in each study population were similar. The average age of patients was 66.31 in the AMI group, and 72.54 in the COPD group. Surgery rates were 14.5% in the AMI patients, and 1.17% in the COPD patients. In-hospital mortality rates were 14.94% in the AMI group, and 3.02% in the COPD group. One-year mortality was 27.07% in AMI patients, and 22.48% in COPD patients (Table 1). The results are followed with three comparisons.

**Table 1:** Characteristics of the Study Populations

	AMI (n = 8,961)		COPD (n = 32,755)	
Age in years (mean ± SD)	66.31 ± 13.36		72.54 ± 12.10	
Male	6,457	(72.06%)	23,650	(72.20%)
Aborigines	161	(1.80%)	1,350	(4.12%)
Patients received surgery	1,299	(14.50%)	382	(1.17%)
In-hospital mortality	1,339	(14.94%)	990	(3.02%)
One-year mortality	2,426	(27.07%)	7,362	(22.48%)

AMI = acute myocardial infarction; COPD = chronic obstructive pulmonary disease.

### (1). To the baseline model

First, we compared each logistic model of three methods to the nested baseline model. Table 2 provides the G^2 ^statistic values resulting from comparing the maximized likelihood function of the nested models. The degree of freedom equals the number of parameters entered in the regression model. Some comorbidity variables were dropped in the analysis because they had 0% or 100% prevalence. For example, using information from the index and prior hospitalizations among the AMI patients, myocardial infarction had 100% prevalence and AIDS had 0% prevalence. Thus, the degree of freedom in this model equals 19. All *p *values of the G^2 ^statistics are significant (< 0.0001) in Table 2. The three comorbidity methods significantly improved the fit of the regression models for both in-hospital and 1-year mortality in the two populations. Table 3 provides the c-statistics for each logistic model. Compared with the baseline model, all three comorbidity measures improved the model prediction for having higher c-statistic values.

**Table 2:** G^2 ^statistics indicating the contribution of the comorbidity measures to the nested baseline model

	**G**^**2† **^**(df**^**‡**^**)**			
	
	In-hospital mortality		One-year mortality	
	
	AMI	COPD	AMI	COPD
Baseline model*	N/A(4)	N/A (4)	N/A (4)	N/A (4)
				
**Index hospitalization only**				
Baseline model + Charlson/Deyo	35 (16)	59 (17)	120 (16)	254 (17)
Baseline model + Charlson/Romano	100 (15)	114 (16)	280 (15)	613 (16)
Baseline model + Elixhauser	216 (33)	269 (33)	394 (33)	856 (33)
				
**Index and prior hospitalizations**				
Baseline model + Charlson/Deyo	80 (19)	98 (20)	336 (19)	1106 (20)
Baseline model + Charlson/Romano	129 (19)	144 (20)	428 (19)	1199 (20)
Baseline model + Elixhauser	194 (33)	200 (33)	496 (33)	1290 (33)

AMI = acute myocardial infarction; COPD = chronic obstructive pulmonary disease.

* Variables in the baseline model included age, sex, race, and whether the patient received surgery.

^† ^All p values of the G^2 ^statistics, < 0.0001.

^‡ ^Degree of freedom equals to the number of parameters entered in the regression model. Some comorbidity variables were dropped in the analysis because they had 0% or 100% prevalence.

N/A: Not applicable.

**Table 3:** C statistics indicating the predictability of each logistic regression model

	In-hospital mortality				One-year mortality			
	
	AMI		COPD		AMI		COPD	
	
	c	95% CI	c	95% CI	c	95% CI	c	95% CI
Baseline model*	0.707	(0.695-0.720)	0.697	(0.684-0.710)	0.736	(0.726-0.746)	0.670	(0.664-0.676)
								
**Index hospitalization only**								
Baseline model + Charlson/Deyo	0.712	(0.701-0.726)	0.708	(0.697-0.723)	0.747	(0.738-0.757)	0.681	(0.675-0.687)
Baseline model + Charlson/Romano	0.723	(0.712-0.737)	0.719	(0.707-0.733)	0.759	(0.750-0.769)	0.692	(0.687-0.698)
Baseline model + Elixhauser	0.737	(0.729-0.753)	0.738	(0.729-0.754)	0.767	(0.760-0.778)	0.701	(0.696-0.707)
								
**Index and prior hospitalizations**								
Baseline model + Charlson/Deyo	0.721	(0.712-0.736)	0.718	(0.707-0.733)	0.766	(0.758-0.776)	0.711	(0.705-0.716)
Baseline model + Charlson/Romano	0.729	(0.719-0.743)	0.726	(0.714-0.740)	0.773	(0.765-0.783)	0.714	(0.708-0.719)
Baseline model + Elixhauser	0.736	(0.729-0.752)	0.731	(0.722-0.747)	0.777	(0.770-0.787)	0.716	(0.711-0.723)

AMI = acute myocardial infarction; COPD = chronic obstructive pulmonary disease.

*Variables in the baseline model included age, sex, race, and whether the patient received surgery.

### (2). Across comorbidity measures for the same outcome, population, and data period

Second, we compared three comorbidity methods when applied to the same outcome, population, and data period. Table 3 shows that the Elixhauser models performed best in all conditions. Moreover, when using the index hospitalization only, the Elixhauser models outperformed the Charlson/Deyo models in all bootstrap replications. For example, predicting in-hospital mortality in the AMI patients, when using information from the index hospitalization, the c-statistic of Elixhauser model is 0.737, which is higher than the Charlson/Deyo (0.712) or Charlson/Romano (0.723). The Elixhauser models outperformed the Charlson/Deyo models in all bootstrap replications (0.729-0.753 vs. 0.701-0.726).

### (3). Within the same outcome, population, and comorbidity measure, but across data periods

Third, we compared model discrimination across data periods within the same outcome, population, and comorbidity measure. Since the Charlson/Deyo and the Charlson/Romano models need information from prior hospitalizations, they performed better when using information from the index and prior hospitalizations compared with the index hospitalization only. In predicting 1-year mortality in the AMI patients, the Charlson/Deyo method had higher c-statistic when using information from the index and prior hospitalizations (0.766) than the index hospitalization only (0.747).

However, the Elixhauser models showed different patterns between different outcomes. In predicting in-hospital mortality, the Elixhauser models performed better when using the index hospitalization only. More importantly, in predicting in-hospital mortality, Table 3 presents further evidence that the Elixhauser models using the index hospitalization only performed best, even better than the Charlson/Deyo or the Charlson/Romano models using the index and prior hospitalizations. In the COPD patients, the Elixhauser method had higher c-statistic when using information from the index hospitalization (0.738) than the index and prior hospitalizations (0.731). Moreover, it is better than the Charlson/Deyo models using the index and prior hospitalizations (0.718) as well as the Charlson/Romano models (0.726).

Nevertheless, in predicting 1-year mortality, the Elixhauser models using information from the index and prior hospitalizations performed better than using information from the index hospitalization only.

## Discussion

To our knowledge, this is the first study that validates the Elixhauser method and compares it to other methods in the Asia region. Further, it is the only investigation that examines the modeling performance of both in-hospital and 1-year mortality of the Elixhauser method, and is the first to report its differences in data periods between short- and long-term outcomes.

Several implications for risk adjustment can be drawn from this study. First, this study showed further evidence of external validity of the Elixhauser method in a different dataset and population. In every comparison, it was superior to the Deyo et al. version of the Charlson comorbidity index, which has been used widely for outcome and epidemiology studies. It also outperformed the Romano et al. adaptation of the Charlson index, which has been reported to be better than the Charlson/Deyo method in several studies [13,15-17]. These findings add to the literature by providing new evidence on the comparison of these three methods together.

Second, our findings expand upon the results of two earlier studies which used the same analytical method in creating comorbidity variables[6,7], and demonstrate the same results not only for short-term mortality but also for long-term mortality. Stukenborg et al. [7] concluded that the Elixhauser method had better statistical performance than the Charlson/Deyo method in predicting in-hospital mortality by creating comorbidity variables as individual categories (the presence or absence of the comorbidity) using California claims data [7]. The same approach was implemented in Canadian administrative data for predicting MI inpatient's mortality, and reported similar findings [6]. Our study suggests that similar results can also be found in predicting 1-year mortality of patients with AMI and COPD. However, the Charlson method may still be a useful tool in many studies because it provides weighted scores, which is valuable if there are insufficient cases to use independent categories for modeling.

Third, new findings from different data periods provide additional insight into the comorbidity measure. Since some diagnoses are included only when they appear in the prior admissions when using the Charlson/Deyo method and the Charlson/Romano method [13,30], these two methods have worse performance using the index hospitalization only. Some disease categories, such as congestive heart failure, never present if there is no prior information [12,13]. This is more important for long-term mortality since the Charlson/Deyo method using the index and prior hospitalizations performed better than using only the index hospitalization in 100% of the bootstrap replications. However, the Elixhauser method develops DRG screen, so it can include more secondary diagnoses, and also distinguishes comorbidities from complications [18]. Our findings agree with previous reports [7] that the Elixhauser method using the index hospitalization performed better than the Charlson/Deyo method using the index and prior hospitalizations when predicting in-hospital mortality. Furthermore, similar results were found when comparing to the Charlson/Romano method in our study. New findings showed that the Elixhauser method has different patterns between different outcomes. Prior information may be more important when predicting 1-year mortality. This may be because only the influential or important diagnoses for the index hospitalization are likely to be recorded, but some conditions that are not directly related to the index hospitalization may be important to the long-term survival.

One limitation of this study is that the administrative data are claimed for reimbursement purposes rather than research purposes and thus varied in data quality [31]. The quality of administrative data depends on the gaps in clinical information, coding procedures, and the billing context [32]. Another limitation is that only two populations were investigated. However, we examined one acute condition and one chronic condition with diverse in-hospital mortality, and found similar results.

Since comparative performance of different comorbidity measures can only be examined when other factors, such as population, outcome, and data periods, are all the same. So does the analysis of data periods. The present study examined three types of comparisons by using a manageable design which focused on three measures of comorbidities, two data periods, two diagnoses, and two outcomes. Moreover, such design could become strength for its simplicity to be applied to other populations in Asia or other areas of the world. Similar studies of comparative performance are needed and can be applied to different populations, datasets, outcomes, data periods, and other case-mix methods.

## Conclusion

In the AMI and COPD patients, the comorbidity measurement developed by Elixhauser et al. has good predictive validity, and researchers should consider its use with claims-based studies rather than the customarily used Charlson/Deyo method. In predicting in-hospital mortality, the Elixhauser method has better discrimination using the index hospitalization; while in predicting 1-year mortality, it may perform better using information from the index and prior 1-year hospitalizations.

## Abbreviations

AHRQ: Agency for Healthcare Research and Quality; AMI: Acute myocardial infarction; CCI: Charlson comorbidity index; CCS: Clinical classifications software; COPD: Chronic obstructive pulmonary disease; DRG: Diagnosis-related group; HCUP: Healthcare Cost and Utilization Project; ICD-9-CM: International Classification of Disease, 9th Revision, Clinical Modification; ROC: Receiver operating characteristic.

## Competing interests

The authors declare that they have no competing interests.

## Authors' contributions

YTC contributed to the study design, statistical analysis, interpretation of data, and writing of the manuscript. YYN contributed to the interpretation of data, and revised the manuscript. SCW contributed to the study design, interpretation of data, and revised the manuscript. All authors have read and approved the final version of the manuscript.

## Pre-publication history

The pre-publication history for this paper can be accessed here:

http://www.biomedcentral.com/1472-6963/10/140/prepub

## Supplementary Material

Additional file 1 **Appendix 1**. Comparison of the components of the models Click here for file
