# Supplementary material for: Comparison of different comorbidity measures for use with administrative data in predicting short- and long-term mortality
Source: BMC Health Serv Res. 2010 May 27;10:140. doi: 10.1186/1472-6963-10-140 (PMC2897792; doi:10.1186/1472-6963-10-140)
Supplement: Additional file 1 — Appendix 1. Comparison of the components of the models [file 1472-6963-10-140-S1.DOC]

**APPENDIX 1.** Comparison of the components of the models

| Variables | Baseline model | Baseline model + Charlson/Deyo | Baseline model + Charlson/Romano | Baseline model + Elixhauser |
| --- | --- | --- | --- | --- |
| Age | ˇ | ˇ | ˇ | ˇ |
| Sex | ˇ | ˇ | ˇ | ˇ |
| Race | ˇ | ˇ | ˇ | ˇ |
| Whether the patient received surgery | ˇ | ˇ | ˇ | ˇ |
| Comorbidities |  |  |  |  |
| Myocardial infarct |  | ˇ | ˇ |  |
| Congestive heart failure |  | ˇ | ˇ | ˇ |
| Peripheral vascular disease |  | ˇ | ˇ | ˇ |
| Cerebrovascular disease |  | ˇ | ˇ |  |
| Dementia |  | ˇ | ˇ |  |
| Chronic pulmonary disease |  | ˇ | ˇ | ˇ |
| Rheumatologic disease |  | ˇ | ˇ | ˇ |
| Ulcer disease |  | ˇ | ˇ |  |
| Mild liver disease |  | ˇ | ˇ |  |
| Diabetes |  | ˇ | ˇ | ˇ |
| Hemiplegia or paraplegia |  | ˇ | ˇ |  |
| Renal disease |  | ˇ | ˇ |  |
| Diabetes with chronic complications |  | ˇ | ˇ | ˇ |
| Any malignancy, including leukemia  and lymphoma |  | ˇ | ˇ |  |
| Moderate or severe liver disease |  | ˇ | ˇ |  |
| Metastatic solid tumor |  | ˇ | ˇ | ˇ |
| AIDS |  | ˇ | ˇ | ˇ |
| Cardiac arrhythmias |  |  |  | ˇ |
| Valvular disease |  |  |  | ˇ |
| Pulmonary circulation disorders |  |  |  | ˇ |
| Hypertension |  |  |  | ˇ |
| Paralysis |  |  |  | ˇ |
| Other neurological disorders |  |  |  | ˇ |
| Hypothyroidism |  |  |  | ˇ |
| Renal failure |  |  |  | ˇ |
| Liver disease |  |  |  | ˇ |
| Peptic ulcer disease excluding  bleeding |  |  |  | ˇ |
| Lymphoma |  |  |  | ˇ |
| Solid tumor without metastasis |  |  |  | ˇ |
| Coagulopathy |  |  |  | ˇ |
| Obesity |  |  |  | ˇ |
| Weight loss |  |  |  | ˇ |
| Fluid and electrolyte disorders |  |  |  | ˇ |
| Blood loss anemia |  |  |  | ˇ |
| Deficiency anemia |  |  |  | ˇ |
| Alcohol abuse |  |  |  | ˇ |
| Drug abuse |  |  |  | ˇ |
| Psychoses |  |  |  | ˇ |
| Depression |  |  |  | ˇ |
